# Characterizing breast conditions at an open-access breast clinic in South Africa: a model that is more than cancer care for a resource-limited setting

**DOI:** 10.1186/s12913-016-1959-4

**Published:** 2017-01-21

**Authors:** Sarah Rayne, Naomi Lince-Deroche, Cheryl Hendrickson, Kate Shearer, Faith Moyo, Pam Michelow, Grace Rubin, Carol Benn, Cynthia Firnhaber

**Affiliations:** 1 Helen Joseph Breast Care Clinic, Helen Joseph Hospital, Johannesburg, South Africa; 2 Department of Surgery, School of Clinical Medicine, Faculty of Health Sciences, University of the Witwatersrand, Johannesburg, South Africa; 3 Health Economics and Epidemiology Research Office, Department of Internal Medicine, School of Clinical Medicine, Faculty of Health Sciences, University of the Witwatersrand, Johannesburg, South Africa; 4 Cytology Unit, National Health Laboratory Service and Department of Anatomical Pathology, Faculty of Health Sciences, University of the Witwatersrand, Johannesburg, South Africa; 5 Department of Radiology, School of Clinical Medicine, Faculty of Health Sciences, University of the Witwatersrand, Johannesburg, South Africa; 6 Clinical HIV Research Unit, Department of Internal Medicine, Faculty of Health Sciences, University of Witwatersrand, Johannesburg, South Africa; 7 Right to Care, Johannesburg, South Africa

**Keywords:** Breast cancer, Open access, Global health, Benign breast disease, Diagnosis

## Abstract

**Background:**

While most breast-related research focuses on cancer, presentation of symptomatic persons in non-screened environments requires understanding the spectrum of breast diseases so as to plan services in resource-constrained settings. This study presents the variety of breast disease managed at a government, open-access breast clinic in South Africa.

**Methods:**

We performed a retrospective file review using a systematic random sample of patients 18 years and above presenting for breast care over a 14-month period. We collected demographics, clinical characteristics, management and final diagnoses from the first visit and twelve subsequent months.

**Results:**

The final sample contained 365 individuals (97 · 5% women). Most were black, unmarried and South African citizens with a median age of 43 years (IQR 31–55) . Of those reporting their status (24 · 1%) 38 · 6% were HIV-positive. A mass (57 · 0%) and/or pain (28 · 5%) were the most common symptoms. Imaging and breast biopsies were required in 78 and 25% of individuals, respectively. Nearly half of biopsies identified breast cancer (44 · 1% of women ≤40 and 57 · 3% for women >40). Benign conditions (47 · 7%) and no abnormality (18 · 2%) were common final classifications among women. There was no difference between the final classifications of patients who self-referred versus those who were formally referred from another health care provider. Nearly half of the participants (46 · 6%) travelled 20 km or more to attend the clinic.

**Conclusions:**

Benign breast conditions far outweighed cancer diagnoses. As breast cancer awareness increases in resource-limited countries, facilities offering breast care require administrative and clinical preparation to manage a range of non-cancer related conditions.

## Background

An estimated 14 million new cancer cases occurred in 2014 worldwide [1]. More than 50% of these occurred in countries with a low or medium-level Human Development Index (HDI) [2], and for women the most common cancers diagnosed were of the breast and cervix [1]. A recent review of breast cancer in Sub-Saharan Africa found that the burden of cancer in this region is growing, with estimates suggesting that age-specific incidence and mortality rates are rising in some countries [3]. According to the most recent data available from South Africa’s National Cancer Registry, in 2009 breast cancer was the leading form of cancer among women in South Africa with an age-adjusted incidence rate of 24 · 1 per 100,000 persons, corresponding to a lifetime risk of 1 in 33 [4]. In 2013, Statistics South Africa reported that breast cancer was the ninth leading cause of death among females aged 45–64 years old, and 65 and older in Gauteng Province, accounting for 3 · 1 and 2 · 1% of deaths, respectively [5].

The significant cancer burden in low or medium-level HDI countries like South Africa impacts on families and communities as well as national-level productivity indicators [2, 6]. Further, a focus on prevention and treatment of cancer often masks the separate problem of other breast disease and the human and economic costs of these conditions. As the medical community and policy makers encourage women to become breast aware, examine their breasts regularly and consider population-level policies for screening for cancer, the health system should be prepared for the diagnosis and management of additional benign breast disease and previously undiagnosed breast problems.

There is global consensus that the diagnosis or exclusion of breast cancer requires a triple assessment [7]. This gold-standard technique of clinical assessment, breast imaging and pathological diagnosis (where indicated) ensures high sensitivity in detection or exclusion of breast cancer [8]. This approach to screening is best employed worldwide in “comprehensive cancer clinics,” or “one-stop clinics” [9]. In South Africa and other countries, the public health system is arranged around primary health clinics, with this first tier of health care serving as the encouraged entry point for patients, who will then move up or down through a system of referral facilities as their disease process dictates. While this model serves many conditions, patients may incur multiple direct and indirect delays through their course to diagnosis and/or treatment [10].

There is limited literature on the provision of breast care services in resource—limited environments—beyond those offered for breast cancer. Further, there is limited information regarding the prevalence of benign breast disease in these settings, particularly in Sub-Saharan Africa, with few studies reporting benign breast conditions in addition to malignant breast disease [11–13]. In this study, we describe the primary complaints and diagnoses of a patient population attending a large, outpatient breast clinic operating within a government hospital in Johannesburg, South Africa. The clinic allows “walk-ins”, or self-referral, directly to tertiary-level services. This allows for exploration of an alternative model for comprehensive breast care management.

## Methods

### Study design, participants and sampling

We conducted a retrospective review of medical records. Patients were eligible for inclusion in the study if they were 18 years or older, had their first visit between 1 April 2011 and 30 June 2012, and had a file available for review at the time of sampling (August-September 2013). Staff at the clinic list all patients attending the clinic for the first time in a “first visit register”. We used this register to obtain a full listing of patients who had attended during the period of interest and to assess the first two eligibility criteria: age and timing of first visit to the clinic. The resulting group of individuals constituted our initial sampling frame.

We selected files for potential participants using a random starting point established using SAS (SAS Institute Inc., Care, NC, USA, Version 9.3) and a pre-determined, fixed sampling interval to reach our desired sample size of 400. Sampling was done separately for men and women to maintain similar proportions to the proportions of patients who had attended the clinic during the period of interest. Based on the number of eligible men and women in the initial sampling frame and our desired sample size, we established a sampling interval (K) of 8 for both genders (e.g. for men the formula was: K = number of men in initial sampling frame ÷ (proportion of men in initial sampling frame x 400)). To ensure an even distribution by age in the final samples of men and women, we sorted the listing of eligible files in the initial sampling frame by age before sampling. Then we selected every K^th^ participant to create an enrolment list and searched for his/her file in the filing room at the clinic. After going through the entire enrolment list, if we had not reached the desired sample size of 400 due to missing files at the facility, the missing files were replaced by drawing additional participants from the sampling frame using the pre-set interval.

### Data collection and analysis

Data collection was conducted from August 2013 to June 2014. For each patient file selected during sampling, trained staff reviewed the file plus any additional, breast care-related records stored separately at the clinic. The team used a standardized data collection form to transcribe information from the records pertaining to: demographics, personal history, surgical/medical history, family history, presenting complaint and/or physical examination findings, radiology, pathology, surgery, chemotherapy, radiation, endocrine, metastases and follow-up visits. During an initial visit to the clinic, the clinic staff had conducted a socioeconomic assessment to determine whether the individual was liable to pay a fee for services and documented this in the file. This information was also collected for the study. The income assessment was converted to US dollars using an average exchange rate for the sampling period of 8 · 2901 South African rands per dollar [14]. Whether the patient was referred in or “self-referred” was determined based on the presence or absence of a referral letter from another facility or health care provider. The absence of such a letter was seen to indicate self-referral. Finally, HIV status was determined through test results, self-report or treatment notes. It was not systematically asked during the intake visit, nor was testing systematically conducted at the facility. Distance travelled to the clinic was calculated from the suburb of residence using Google maps shortest recommended journey time in private car. These may be conservative estimates of actual distances travelled.

Data was collected from the first documented visit through 12 months of follow-up for each patient. The clinic staff follow a diagnosis algorithm which includes conducting a physical exam with all first-visit clients. Those individuals who are determined to have no abnormalities are given a date for mammography if they are over 40 years old and have not had a mammogram in the last year. If under 40, they are told to return to the clinic after a year for a check-up. For anyone who is not diagnosed as “normal” at the physical exam, the staff will request imaging and/or histological investigation as necessary. Patients with abnormal imaging and/or histological results may require further investigation to confirm or exclude malignancy, and therefore might require multi-day appointments.

We used REDCap (hosted by the University of the Witwatersrand) to enter and manage the data [15], and we analysed the data using SAS (SAS Institute, Cary, NC, version 9.3). We explored and present demographic characteristics by sex. We also present primary complaints and results of the triple assessment (i.e. physical exams, radiology and histology procedures) by age (stratified across two tables by sex). We analysed demographic characteristics using medians for continuous variables (due to non-normal distribution) and frequencies for categorical variables.

Approval for the study was granted by the Human Research Ethics Committee of the University of Witwatersrand in Johannesburg, South Africa and the Chief Executive Officer at the hospital where the study took place.

## Results

**Fig. 1:**
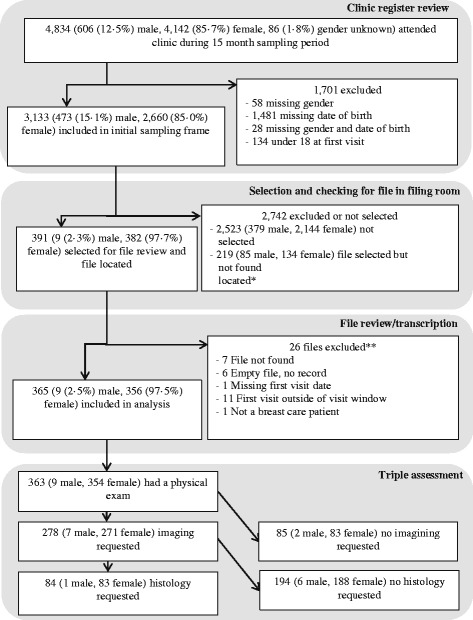
Selection and inclusion of files for review and presentation of diagnostic pathway for selected files

Figure 1 illustrates the selection and inclusion process. During the sampling-eligible period (1 April 2011 to 30 June 2012), 4,834 individuals attended the clinic. The population attending services at the clinic was mostly female (4,142, 85 · 7%); though a substantial number of men also attended (606, 12 · 5%). Sex was unknown for 86 (1.8%) clinic attendees.

All individuals with missing date of birth (*n* = 1,510), those who were under 18 (*n* = 134), and those whose sex had not been recorded were excluded. Ultimately, 3,133 individuals were eligible for inclusion in the initial sampling frame. We selected 610 files (94 (15%) men, 516 (85%) women) from the sampling frame; nine (9 · 5%) of the selected men’s files could be located and 382 (74%) of the women’s. Thus, a total of 391 individuals were enrolled into the study. During the review and transcription process, an additional 26 files (all female) were excluded due to errors found in the initial visit register indicating study ineligibility or the inability to locate the files for transcription. As a result, 365 files (356 women, 9 men) were included in the review.

Demographic and clinical characteristics for the individuals included in the file review are presented in Table 1. The majority of the participants were black (51 · 5%), unmarried (61 · 4%) and South African citizens (60 · 5%). Considering all patients — male and female — the median age was 43 years [Interquartile Range (IQR): 31–55]. However, the female patients tended to be younger with 44.7% presenting under age 40 years compared to 22.2% of males. HIV status was available for just over 24% of the study population (*n* = 88). Considering those for whom status was known, 34 (38 · 6%) were known to be HIV-positive. However, these 34 HIV-positive individuals represent just 9.3% of the entire study population.

**Table 1:** Demographic and clinical characteristics of patients presenting for care (*n* =365)

Characteristic	Total (*n* = 365)		Female (*n* = 356)		Male (*n* = 9)	
	*n*	% (IQR)	*n*	% (IQR)	*n*	% (IQR)
Age						
≤40	161	44 · 1	159	44 · 7	2	22 · 2
40–55	113	31 · 0	110	30 · 9	3	33 · 3
>55	91	24 · 9	87	24 · 4	4	44 · 4
Median	43	(31–55)	43	(30–55)	48	(35–56)
Marital status						
Single	165	45 · 2	161	45 · 2	4	44 · 4
Married	125	34 · 2	122	34 · 3	3	33 · 3
Divorced/separated/widowed	59	16 · 2	57	16 · 0	2	22 · 2
Not reported/missing	16	4 · 4	16	4 · 5	0	0 · 0
Race						
Black	188	51 · 5	181	50 · 8	7	77 · 8
White	36	9 · 9	36	10 · 1	0	0 · 0
Coloured^a^	23	6 · 3	22	6 · 2	1	11 · 1
Asian	12	3 · 3	12	3 · 4	0	0 · 0
Other	16	4 · 4	16	4 · 5	0	0 · 0
Not reported/missing	90	24 · 7	89	25 · 0	1	11 · 1
Nationality						
South African	221	60 · 5	216	60 · 7	5	55 · 6
Non-South African	19	5 · 2	18	5 · 1	1	11 · 1
Not reported/missing	125	34 · 2	122	34 · 3	3	33 · 3
HIV status^b^						
Positive	34	9 · 3	29	8 · 1	5	55 · 6
Negative	54	14 · 8	52	14 · 6	2	22 · 2
Unknown	277	75 · 9	275	77 · 2	2	22 · 2
Family history of cancer						
Breast	45	12 · 3	44	12 · 4	1	11 · 1
Other	14	3 · 8	14	3 · 9	0	0 · 0
None	149	40 · 8	148	41 · 6	1	11 · 1
Not reported/missing	157	43 · 0	150	42 · 1	7	77 · 8
Medical aid/income assessment^c^						
No medical aid/Unemployed, receiving pension or grant	55	15 · 1	55	15 · 4	0	0 · 0
No medical aid/Income <R4,000	276	75 · 6	267	75 · 0	9	100 · 0
No medical aid/ Income >R4,000	10	2 · 7	10	2 · 8	0	0 · 0
Has private medical aid	22	6 · 0	22	6 · 2	0	0 · 0
Not reported	2	0 · 5	2	0 · 6	0	0 · 0
Distance to facility (km)						
<5	49	13 · 4	47	13 · 2	2	22 · 2
5–19	129	35 · 3	126	35 · 4	3	33 · 3
20–60	148	40 · 5	145	40 · 7	3	33 · 3
>60	22	6 · 0	22	6 · 2	0	0 · 0
Unknown/missing	17	4 · 7	16	4 · 5	1	11 · 1
Origin of referral						
Self-referral	258	70 · 7	251	70 · 5	7	77 · 8
Referral from healthcare provider	107	29 · 3	105	29 · 5	2	22 · 2
Number of exams/procedures during 12 month study collection period						
1	150	41 · 1	145	40 · 8	5	55 · 6
2	162	44 · 4	159	44 · 7	3	33 · 3
3 or more	53	14 · 5	52	14 · 5	1	11 · 1

^a^The term “coloured” is South Africa’s official determination for individuals of mixed-race

^b^As determined through documentation of test result, self-reported status or documentation of currently taking antiretroviral medication

^c^Categories for socioeconomic status as assessed by clinic staff. R4,000 is equivalent to US$482.50

Three-quarters of all patients fell into the “No medical aid/ Income <R4,000 (US $482.50)” bracket during the socioeconomic assessment. Almost half of the participants (46 · 6%) had travelled 20 km or more to get to the clinic; their mode of transport was unknown. Most (70 · 7%) were “self-referred” or walked in on the intake day without a referral from another facility, and 41 · 1% had only one visit to the study clinic.

**Fig. 2:**
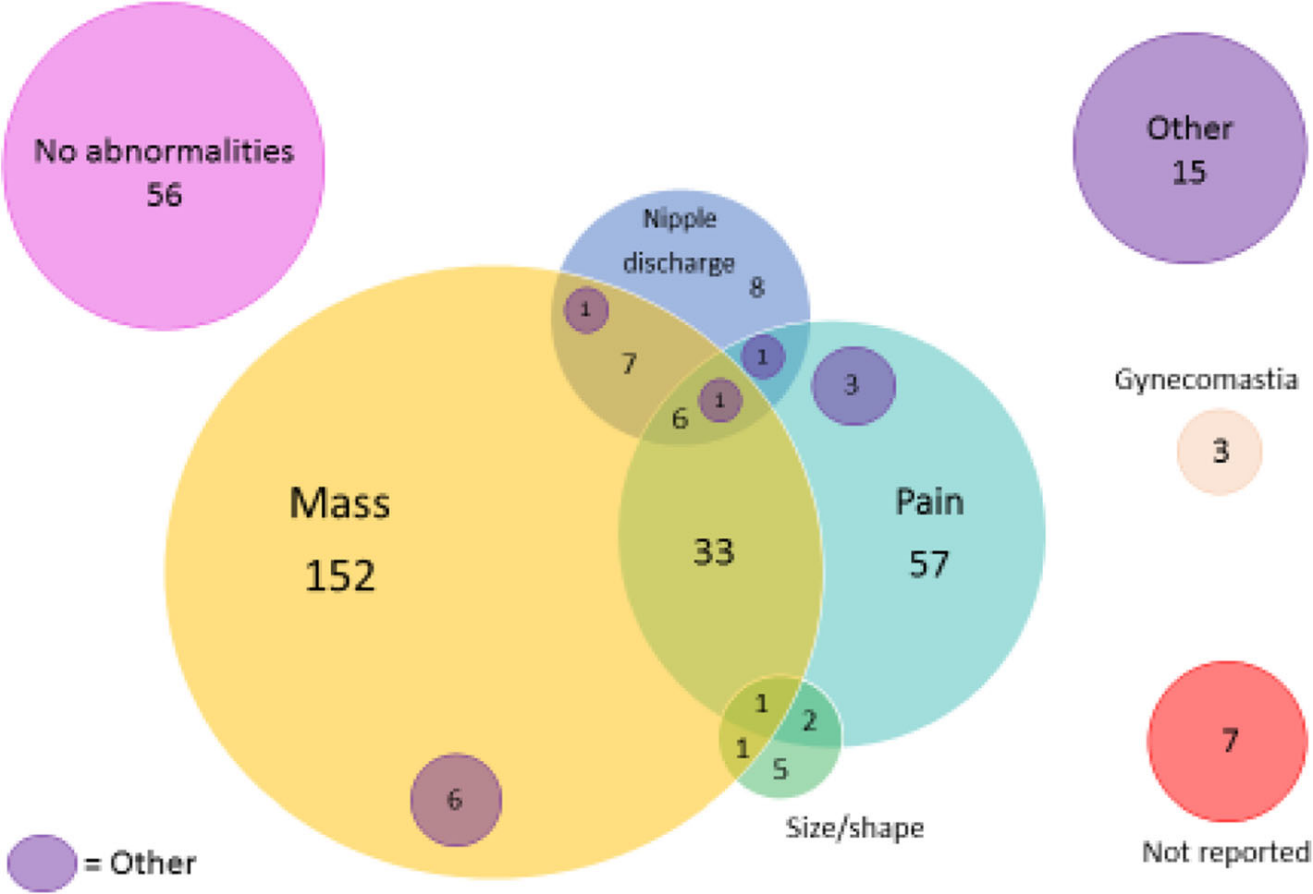
Recorded complaint from patients presenting for care (*n* = 365)

Figure 2 illustrates the initial classification of patients based on a reported complaint or assessment during the physical exam at the intake visit. Mass (57 · 0%), pain (27 · 7%), discharge (6 · 6%) and size/shape issues (2 · 5%) were common; often an individual had more than one complaint or finding. A notable number of patients were also found during the exam to have no abnormalities (15 · 3%). Among the nine who were noted to have had the complaint and/or diagnosis of “size/shape,” three requested a reduction, two had asymmetry and the remaining patients had other aesthetic concerns. Both reduction and augmentation procedures are done at the facility; however the augmentations are done on an extremely limited basis and for surgical training purposes only. The three isolated gynecomastia cases were found in men, all of whom were HIV positive on antiretroviral therapy.

We present the results of the clinic’s triple assessment algorithm by sex in Table 2. For women the results are split by age (≤40 years old versus >40). The initial exam results were similar for women in the two age categories. Considering all imaging procedures performed for women, roughly three quarters identified an abnormality (77 · 5% of women ≤40 and 68 · 1% for women >40). Further, considering histological procedures done among women, nearly half identified a breast cancer (44 · 1% of women ≤40 and 57 · 3% for women >40). Benign conditions were the most common final classification among women. This included fibroadenoma (15.1%), breast pain (14 · 2%), duct ectasia (3 · 4%) and papilloma (<1%). For men, gynecomastia (66 · 7%) was the most common final classification.

**Table 2:** Triple assessment procedural results by sex and age (for women only) and final classification^a^

Result	Women (*n* = 356)				Men (*n* = 9)	
	≤40		>40			
	*n*	%	*n*	%	*n*	%
Clinical exam result	*n* = 159		*n* = 197		*n* = 9	
Mass only	66	41 · 5	83	42 · 1	3	33 · 3
Pain only	26	16 · 4	30	15 · 2	1	11 · 1
Discharge only	6	3 · 8	2	1 · 0	0	0 · 0
Other only	9	5 · 7	6	3 · 0	0	0 · 0
Gynecomastia only	0	0 · 0	0	0 · 0	3	33 · 3
Size/shape issues only	2	1 · 3	3	1 · 5	0	0 · 0
More than one of the complaints listed above	27	17 · 0	34	17 · 3	1	11 · 1
No abnormalities/routine check-up	19	11 · 9	36	18 · 3	1	11 · 1
Not reported/inconclusive	4	2 · 5	3	1 · 5	0	0 · 0
Radiological/imaging tests requested	*n* = 131		*n* = 273		*n* = 7	
Results available	111	84 · 7	229	83 · 9	4	57 · 1
Mass/density/distortion	62	55 · 9	85	37 · 1	0	0 · 0
Micro-calcification	2	1 · 8	27	11 · 8	0	0 · 0
Abscess	6	5 · 4	2	0 · 9	0	0 · 0
Cyst	4	3 · 6	18	7 · 9	1	25 · 0
Other	12	10 · 8	24	10 · 5	1	25 · 0
No abnormalities	23	20 · 7	70	30 · 6	2	50 · 0
Not reported/inconclusive	2	1 · 8	3	1 · 3	0	0 · 0
Results not found	20	15 · 3	44	16 · 1	3	42 · 9
Pathological tests requested	*n* = 41		*n* = 95		*n* = 1	
Results available^b^	34	82.9	82	86.3	1	100.0
Breast cancer (any biopsy including breast and axilla)	15	44 · 1	47	57 · 3	0	0 · 0
Fibroadenoma	2	5 · 9	5	6 · 1	0	0 · 0
Papilloma	3	8 · 8	7	8 · 5	0	0 · 0
Other	13	38 · 2	19	23 · 2	0	0 · 0
Normal/no abnormalities	1	2 · 9	3	3 · 7	1	100 · 0
Not reported/inconclusive	0	0 · 0	1	1 · 2	0	0 · 0
Results not found	7	17 · 1	13	13 · 7	0	0 · 0
Final classification (*n* = 357)	*n* = 159		*n* = 197		*n* = 9	
Cancer	10	6 · 3	42	21 · 3	0	0 · 0
Benign	76	47 · 8	82	41 · 6	1	11 · 1
Infection	12	7 · 5	5	2 · 5	0	0 · 0
Gynecomastia	0	0 · 0	0	0 · 0	6	66 · 7
Plastics	13	8 · 2	5	2 · 5	0	0 · 0
Other^b^	9	5 · 7	8	4 · 1	0	0 · 0
Normal	29	18 · 2	49	24 · 9	1	11 · 1
Not reported/inconclusive	10	6 · 3	6	3 · 0	1	11 · 1

^a^Figure 1 indicates the number of individuals who had a physical exam, imaging and/or histology. In contrast, the information presented here reflects the results for all diagnostic procedures done. Some participants had more than one radiological or histological test

^b^Other consists of the following: mass (unspecified), papilloma, benign neurofibroma

**Fig. 3:**
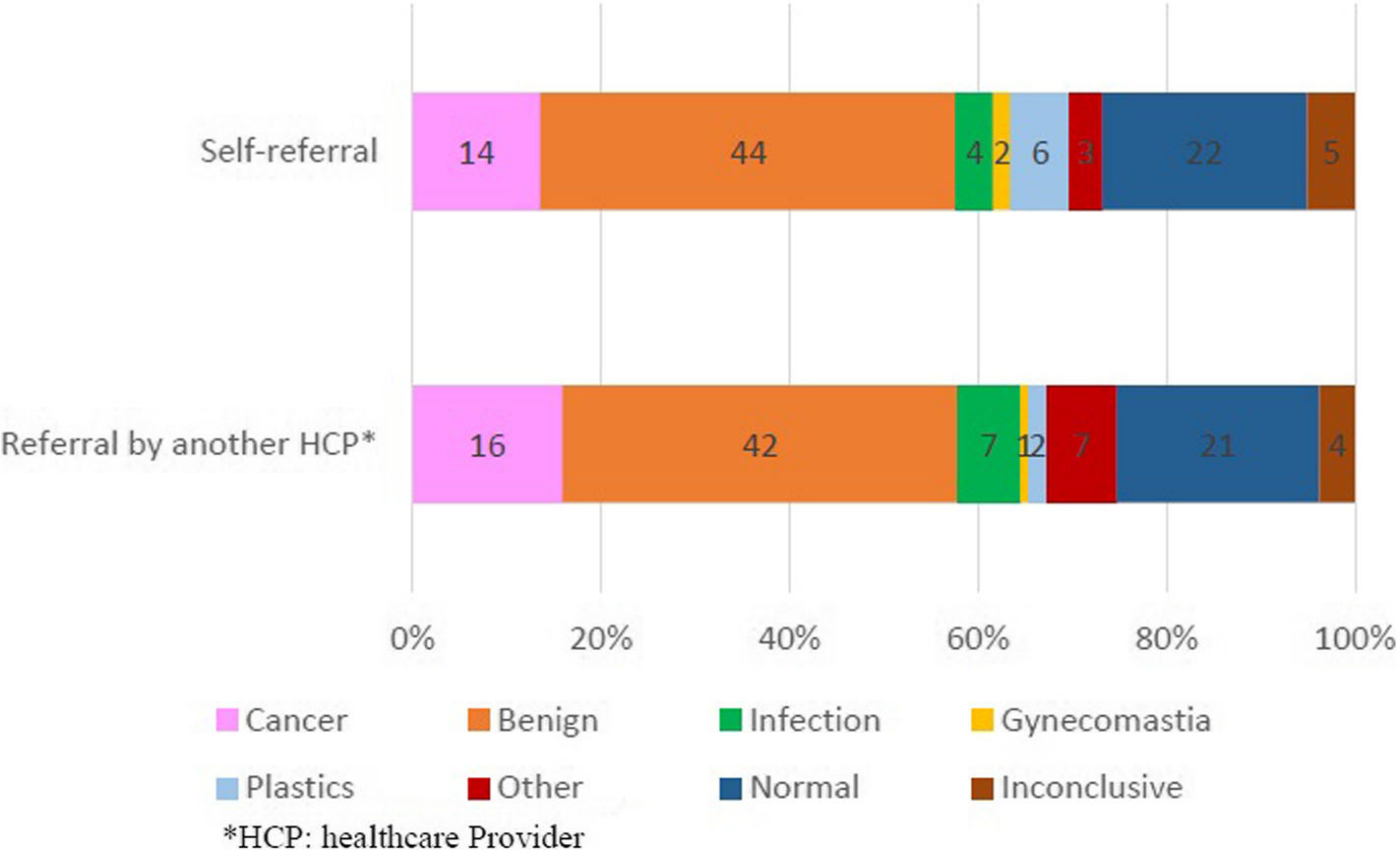
Final classification by origin of referral (*n* = 365)

Figure 3 illustrates the final classification, or diagnosis, for patients who were referred (*n* = 107) to the study facility versus those that self-referred (*n* = 258), or walked-in. The diagnosis of cancer is similar in the two groups (15 · 9 vs 13 · 6%).

## Discussion

During the study period nearly 5,000 new patients attended the breast clinic. When considering the 365 files reviewed, the majority of patients presenting at this breast clinic did not have breast cancer, and most were managed either for benign conditions or self-initiated screening. Only 14% of the patients included in the file review were diagnosed with breast carcinoma or other cancers of the breast. In contrast, more than one third of patients required management of benign breast pathology, and this increased to more than 50% if pain was also included. These findings are similar to those in other low or medium-level HDI countries, where findings of fibroadenoma and fibrocystic change occur far more commonly than malignancy in clinico-pathological studies [11, 12, 16–18], and in studies of symptomatic undiagnosed patients where incidence of cancer was less than 10% [19]. These cancer incidence rates differ from a recent study at Rwanda’s first public cancer referral center which found higher cancer incidence, with 45.3% of presenting patients diagnosed with a breast malignancy. Investigators on the study note that this higher detection rate may be due referral patterns and decision-making in the context of a relatively new breast screening, evaluation and referral system [13]. Of note, not discussed in the predominantly clinico-pathological studies, is a high incidence of breast pain alone (16 · 4% in our sample, and 11% in a Pakistan study [18]) seen and treated in clinic patients. It may be that as referral systems and breast evaluation capacity increases the incidence of benign disease and presentation with breast pain my increase in these settings.

Patients in this study were typically young, black and South African, with demographics reasonably well-matched to the urban environment indicated as the hospital’s catchment area by South Africa’s census data [5]. However, we saw that more than 47% of patients had traveled 20 km or more to access the clinic. In this urban part of the country, this would typically involve bypassing at least one other hospital and numerous primary clinics, indicating a willingness by patients to be seen at a specialist center. This phenomenon of preference for direct access to a specialist has been described in other environments such as within managed healthcare systems [20] or related to extremely specialized care, where a generalist or primary care appointment may not add value [21, 22] and may delay onset of treatment [9, 22, 23].

However, in this study, the ability and/or desire to travel for specialist care must be considered in light of the patients’ income levels. Most reported monthly income at or below R4,000 per month (roughly US$ 482 per month or US$ 5,790 per year) during the study period (April 2011-June 2012). Minimum wage information for South Africa is dependent on one’s industry and job function, but the minimum monthly wage for a domestic worker during 2014, for example, was R2,065 (US$ 249 per month) [24]. In South Africa, in theory, breast care is provided in every secondary or tertiary hospital under the auspices of general surgeons. The spending of limited wages on travel to a specialist breast clinic may underscore the limited availability or limited knowledge of the availability of breast care services — on the part of referring providers and/or patients. It may also indicate a preference by patients for the open-access and/or holistic approach to care provided at this clinic. Additionally, distance travelled to the clinic did not differ between age categories. Interestingly we found there was no difference between the final diagnostic classification of patients who self-referred versus those who had been formally referred from another health care provider or facility. This would indicate that an additional layer of healthcare provider interaction did not improve the sensitivity of differentiation between benign or malignant disease.

In our study almost 65% of the patients were “self-referred” to the clinic. This was even more common among the male patients (at more than 77%). Systems requiring referral can cause delays in the diagnosis and treatment pathway [23]. This could be the result of failure to refer by the staff at the point of entry into the health system, delay in appointment times at specialist clinics, and the myriad logistical and economic problems associated with making many visits to health care facilities [23]. In contrast, open access clinics reduces access times and has been shown in some instances to increase satisfaction and reduce healthcare costs in primary and hospital healthcare [25]. In breast cancer, delays to time to treatment impact directly on survival [10, 26]. Therefore, reducing the barriers to treatment, including multiple visits to different tiers of the healthcare system, and the patient costs associated with this, help improve patient care and may reduce mortality [27]. In patients without cancer, an open-access tertiary clinic can either immediately institute investigations or reassure patients by protocol-based stratification of risk based on clinical findings. In our setting, this allowed resolution of problems such as solitary symptoms of cyclical pain or cosmetic issues to be resolved in one visit at the patient’s convenience. Further investigations to confirm or exclude malignancy did require further appointments, but these could be triaged according to risk and social circumstances (with transport and financially at risk patients investigated on the same day) or patients educated to ensure compliance with appointments. The requirement of return for investigations and results is supported by Dey et al. who found that beyond the first 24 h there is no greater dissipation of anxiety between same-day and follow-up diagnosis, at greater cost with the same-day model [9]. In our model we seek to improve access to definitive specialist care through an open access model, but accept that, once in the system, further appointments will be required.

This study has limitations which reflect some of the hurdles of research based on file review in a resource-limited environment and which highlight the need for support of research orientated clinical practices. The filing system at the clinic was paper-based and lacking in terms of controls for initiating a new file and updating older files. This made navigating the files difficult and resulted in an underrepresentation of men in our final sample. Also, the information in the files was often incomplete. It was difficult to assess the exact number of visits made by patients to the study facility; however, the team received special permissions to search for test results from all possible sources. Finally, the absence of a systematic approach for assessing HIV status at the clinic also resulted in unknown HIV status for many of the study participants. In part to address the limitations identified during the study, after the study, the clinic staff instituted new filing practices and an electronic data management system to hopefully facilitate further research in the future. Despite its limitations, this study addresses a current gap in knowledge about breast disease in Africa, especially in areas with high HIV prevalence.

## Conclusions

In this study, we have described the major pathology determined in patients presenting with breast symptoms and highlighted that breast cancer is not the only reason for comprehensive breast care. As we move to an era of proactive cancer awareness in Africa, in recognition of its important community and national impact, we should expect and encourage an increase in the number of patients attending clinics requiring the management of breast disease. Further, with the global movement towards improved recognition of surgical diseases as public health issues it is important to institute mechanisms to cope with the full spectrum of breast disease. We present our model of open-access, walk-in care as a contribution to this process of improved service delivery. As awareness of breast cancer is promoted by national cancer policies, comprehensive breast clinics can have the ability to deal effectively with the ensuing increase in all forms of breast symptoms with the goal of improving healthcare on the continent.

## Abbreviations

HDI: Human development index
IQR: Interquartile range
